# Molecular Imaging: From Bench to Clinic

**DOI:** 10.1155/2014/357258

**Published:** 2014-12-29

**Authors:** Yì-Xiáng J. Wáng, Yongdoo Choi, Zhiyi Chen, Sophie Laurent, Summer L. Gibbs

**Affiliations:** ^1^Department of Imaging and Interventional Radiology, Faculty of Medicine, The Chinese University of Hong Kong, Prince of Wales Hospital, Shatin, Hong Kong; ^2^Molecular Imaging and Therapy Branch, National Cancer Center, 323 Ilsan-ro, Goyang-si, Gyeonggi-do 410-769, Republic of Korea; ^3^Laboratory of Ultrasound Molecular Imaging, The Third Affiliated Hospital of Guangzhou Medical University, Guangzhou 510150, China; ^4^NMR and Molecular Imaging Laboratory, Department of General, Organic and Biomedical Chemistry, Université de Mons, 7000 Mons, Belgium; ^5^Department of Biomedical Engineering, Center for Spatial Systems Biomedicine, Knight Cancer Institute, Oregon Health and Science University, Portland, OR, USA

Advances in molecular biology and new developments in imaging, engineering, and novel contrast agents position molecular imaging to play a major role in disease management. This current issue mainly covers ultrasound techniques and radionuclide imaging techniques, and both preclinical research on animal models and clinical studies on cancer patients are presented herein. Molecular imaging has demonstrated its potential in characterizing disease models in small animal preclinical studies. In the clinical setting, many patients have the potential to benefit from these new and quantitative imaging techniques providing improved disease characterization, therapeutic monitoring, and objective prognostic criteria. To realize the full potential of molecular imaging, chemists, molecular biologist, and biomedical engineers will need to work closely with clinicians to translate molecular imaging techniques into clinical applications, ultimately providing improved disease diagnosis, treatment monitoring, and noninvasive prognostic imaging for patients.

Advances in molecular biology and new developments in imaging, engineering, and novel contrast agents position molecular imaging to play a major role in disease management [1–16]. Ultrasound (US) imaging has great potential in molecular imaging because microbubble agents are nontoxic and can be used at very low dosages. US can reach both superficial and deep tissues depending on the frequency utilized for imaging. US contrast agents can also be targeted and used as carriers for local gene or drug delivery [5–7]. In addition, US is advantageous because it is of low cost and is widely available. Radionuclide imaging holds great promise due to its high sensitivity, with the small doses of radiotracer necessary and minimal background for imaging. Using appropriate tracer radionuclides, positron emission tomography (PET) and single photon emission computed tomography (SPECT), has demonstrated the capacity to image cellular and molecular targets [1, 2, 5, 16, 17]. However, production of radionuclide agents can be complex and expensive with low stability and limited availability. Optical imaging techniques (fluorescence and bioluminescence) are widely used in small animals, and clinical use is on the rise for imaging superficial tissues such as the breast. However, only fluorescence has clinical applicability, while bioluminescence remains a preclinical research tool because of the requirement of expression of luciferase by the genome. Optical imaging techniques are rapidly evolving, where their sensitivity is similar to radionuclide imaging in terms of detection of low concentrations of contrast agent and are advantageous because contrast agents are significantly more stable overtime. Two near-infrared imaging probes, methylene blue and indocyanine green, have been approved by the FDA for clinical use [9]. The main drawback of optical imaging techniques are the high level of attenuation of signal with depth and the background signal due to autofluorescence, calling for new fluorophores to improve* in vivo* imaging [4, 9]. MRI is also a powerful method for molecular imaging, with novel techniques developing rapidly, including MR spectroscopy, chemical shift imaging, diffusion-weighted imaging, T1rho weighted imaging, chemical exchange saturation transfer, and targeted contrast agents [10–17].

There are currently three imaging strategies to noninvasively monitor and measure molecular events. They have been broadly defined as “direct,” “biomarker,” and “indirect” imaging [2, 3]. The “direct” molecular imaging motif builds on established chemistry and radiochemistry relationships. Bioconjugate chemistry can be used to link specific binding motifs and bioactive molecules to imaging agents, such as superparamagnetic particles for MRI or radionuclides for PET and SPECT imaging [5, 12]. However, a constraint limiting direct imaging strategy is the concentration of direct imaging targets necessary for imaging the individual target to enable visualization. Another constraint limiting direct imaging strategy is the necessity to develop a specific probe for each molecular target and then to validate the sensitivity, specificity, and safety of each probe for specific applications before their introduction into the clinic. A biomarker is a biological or biochemical change that is objectively measured as an indicator of biological processes or pharmacological responses to a therapeutic intervention. For “biomarker imaging,” many existing imaging technologies are used for monitoring downstream changes of specific molecular/genetic pathways in diseases. Some biomarker probes could also be classified as direct imaging probes. For example, [^18^F] 2-fluoro-2-deoxyglucose (FDG) can be considered a direct imaging substrate for visualizing hexokinase enzyme levels, as well as a biomarker for imaging, and is useful in a clinical setting for the identification of malignant lesions, for staging the extent of disease and, in some cases, to evaluate the treatment response, for example, in the case of gastrointestinal stromal tumour (GIST)-Gleevec ^18^F-FDG [16–21]. However, biomarker imaging is likely to be less specific and more limited in measuring the activity of a particular “upstream” pathway. “Indirect” molecular (reporter gene) imaging studies will be more limited in patients compared with that in animals due to the necessity of transducing target tissue cells with a specific reporter construct or the production of transgenic animals bearing the reporter construct [2].

This current issue mainly covers ultrasound techniques and radionuclide imaging techniques, and both preclinical research on animal models and clinical studies on cancer patients are presented herein. Contrast-enhanced ultrasound (CEUS) and acoustic radiation force impulse elastography (ARFI) are evaluated in papers of this special issue. In a population of rectal carcinoma patients, Y. Wang et al. report a positive linear correlation between the CEUS enhanced intensity (EI) and microvessel density (MVD) evaluated by immunohistochemical staining of surgical specimens. Additionally, a significant difference for EI histological grading with EI decreased as *T* stage increased was found. The authors concluded that EI of endorectal CEUS provides noninvasive biomarker of tumor angiogenesis in rectal cancer. In another paper, J.-X. Zhang et al. showed CEUS was helpful in identifying BI-RADS category 3 or 4 small breast lesions and improved diagnostic sensitivity, reduced the negative likelihood ratio, and improved the negative predictive value for these lesions. X. Xu et al. evaluated ARFI for liver tumor radiofrequency ablation results assessment and determined that while CEUS accurately detected residual tumors, the ARFI technique has limited capacity to detect residual tumors; moreover, it was demonstrated that liver cirrhosis is associated with decreased chance of a complete ablation. In a review article, Y.-Y. Liao et al. discussed ultrasound targeted microbubble destruction (UTMD) as a gene delivery system, the combination of UTMD and gene therapy or stem cell therapy in angiogenesis research, and outlined the future challenges in the field.

Expression of multidrug pumps including P-glycoprotein (Pgp) in the plasma membrane of tumor cells often results in decreased intracellular accumulation of anticancer drugs causing serious impediment to successful chemotherapy. It has been shown earlier that combined treatment with UIC2 anti-Pgp monoclonal antibody and cyclosporine A is an effective way of blocking Pgp function. In this special issue, G. Trencsényi et al. investigated the suitability of four PET tumor diagnostic radiotracers, namely, ^18^F-FDG, ^11^C-methionine, 3′-deoxy-3′-[18F]-fluorothymidine (^18^F-FLT), and [18F]-Fluoroazomycin-arabinofuranoside (^18^FAZA) for* in vivo *follow-up of the efficacy of chemotherapy in both P-glycoprotein positive (Pgp+) and negative (Pgp−) human tumor xenograft pairs raised in CB-17 SCID mice. It was found that combined treatment resulted in a significant decrease of both the tumor size and the accumulation of the tumor diagnostic tracers in the Pgp+ tumors. These results demonstrated that ^18^F-FDG, ^18^F-FLT, ^18^F-FAZA, and ^11^C-methionine are suitable PET tracers for the diagnosis and* in vivo *follow-up of the efficacy of tumor chemotherapy both in Pgp+ and Pgp− human tumor xenografts by miniPET. In another experimental study, X. Bao et al. demonstrated that early treatment response of sunitinib was monitored in U87MG model mimicking glioblastoma multiforme by longitudinal ^18^F-FLT microPET/CT imaging. In a study by W. Ma et al., a peptide containing Asn-Gly-Arg (NGR) sequence was synthesized and labeled with ^99m^Tc, and its radiochemical characteristics, biodistribution, and SPECT imaging were evaluated in nude mice bearing human HepG2 hepatoma, demonstrating the potential of  ^99m^Tc-NGR for SPECT imaging agent of tumor. In the clinical study by K. Miwa et al., it was suggested that ^11^C-methionine PET is a marker of the biological characteristics of glioblastoma multiforme and is useful for therapy planning of hypofractionated high-dose irradiation by intensity-modulated radiation. Studies by H. D. Zuo et al. report the effect of iRGD peptide (CRGDK/RGPD/EC) combined with SPIO on the labeling of pancreatic cancer cells. Their results describe a simple protocol to label panc-1 cells using SPIO in combination with iRGD peptide and suggested a method to improve the sensitivity of pancreatic cancer imaging.

We hope readers find the progress on molecular imaging reported in this special issue interesting and stimulating. Molecular imaging has demonstrated its potential in characterizing disease models in small animal preclinical studies. In the clinical setting, many patients have the potential to benefit from these new and quantitative imaging techniques providing improved disease characterization, therapeutic monitoring, and objective prognostic criteria [3]. Current progress on clinical translation of targeted molecular imaging agents is less than initially anticipated. To realize the full potential of molecular imaging, chemists, molecular biologist, and biomedical engineers will need to work closely with clinicians to translate molecular imaging techniques into clinical applications, ultimately providing improved disease diagnosis, treatment monitoring, and noninvasive prognostic imaging for patients.

*Yì-Xiáng J. Wáng* *Yongdoo Choi* *Zhiyi Chen* *Sophie Laurent* *Summer L. Gibbs*
